# Effect of CaO on NO_*x*_ Reduction by Selective Non-Catalytic Reduction under Variable Gas Compositions in a Simulated Cement Precalciner Atmosphere

**DOI:** 10.3390/ijerph14121474

**Published:** 2017-11-29

**Authors:** Ye Sun, Weiyi Fan, Tianle Zhu, Xiaowei Hong

**Affiliations:** School of Space and Environment, Beihang University, Beijing 100191, China; suny@buaa.edu.cn (Y.S.); fanweiyi1226@sina.com (W.F.); hongxiao86@126.com (X.H.)

**Keywords:** CaO, NO_*x*_ reduction, SNCR, cement precalciner atmosphere

## Abstract

High-concentration CaO particles and gas compositions have a significant influence on NO_*x*_ reduction by selective non-catalytic reduction (SNCR) in cement precalciners. The effect of gas composition on NO_*x*_ reduction by SNCR with NH_3_ was studied in a cement precalciner atmosphere with and without CaO at 700–1100 °C. It was found that CaO significantly lowers NO_*x*_ reduction efficiency between 750 °C and 1000 °C, which is attributed to the catalytic oxidation of NH_3_ to NO. Although increasing NH_3_ concentration was advantageous to NO_*x*_ reduction, the existence of CaO led to the opposite result at 750–900 °C. Adding H_2_O can suppress the negative effect of CaO on NO_*x*_ reduction. Decreasing O_2_ content from 10% to 1% shifts the temperature range in which CaO has a significant effect from 750–1000 °C to 800–1050 °C. CO has a variety of influences on the CaO effect under different experimental conditions. The influences of NH_3_, H_2_O, O_2_, and CO on the effect of CaO can be attributed to the impacts of the gas compositions on gas-phase NH_3_ conversion, gas-solid catalytic NH_3_ oxidation, or both processes. A proposed pathway for the effect of gas compositions on NO_*x*_ reduction in CaO-containing SNCR process was developed that well predicted the CaO-containing SNCR process.

## 1. Introduction

Air pollution is now one of the most serious environmental problems worldwide. Nitrogen oxides (NO_*x*_, including NO and NO_2_) emitted from cement rotary kilns are major contributors to acid rain, photochemical smog and haze [1]. In China, 1.7 Mt NO_*x*_ was emitted by the cement industry in 2015, accounting for 9.1% of total NO_*x*_ emissions from anthropogenic sources [2]. The cement industry has become the third-largest NO_*x*_ emission source in China, after the thermal power industry and vehicles. Meanwhile, as the largest cement-producing country in the world, “Emission Standard of Air Pollutants for Cement Industry” (GB4915-2013), which is regarded as the strictest in history, has been issued by the Chinese government to reduce NO_*x*_, SO_2_, NH_3_ and Hg^0^ emissions.

Current NO_*x*_ emission control technologies in the cement industry include low-temperature sintering, staged combustion, low NO_*x*_ burners, over-fire air, SNCR and selective catalytic reduction (SCR) [3]. By far, SNCR is the predominant deNO_*x*_ technology in cement rotary kilns due to its suitable, efficient, and cost-effective performance. Additionally, since SNCR is typically conducted in the cement precalciner, where the temperature ranges from 850 °C to 1200 °C and conforms to the temperature window of SNCR, some countries have introduced full-scale SNCR into the cement industry. However, as opposed to SNCR for electric power generation, NO_*x*_ reduction efficiencies have varied from 15% to 80% between projects, and even within individual projects, for cement kilns [4,5]. This variability is mainly due to the complicated environment in the cement precalciner, including temperature, gas composition and Ca-based particles (95% composed of CaO) from raw material calcinations. Therefore, the influences of multiple factors on NO_*x*_ reduction by SNCR in a simulated cement precalciner atmosphere have been researched extensively.

Previous researchers, including our team, have investigated the effects of O_2_, H_2_O, SO_2_, CO, etc. on the SNCR process over various temperature ranges [6,7,8,9]. In addition to temperature and gas compositions, CaO particles of high concentration from raw material calcinations have a significant influence on NO_*x*_ reduction in a cement precalciner. Actually, catalytic reactions involving NH_3_ and NO can occur on the CaO surface [10,11]. To date, researchers have investigated catalytic NH_3_ decomposition, NH_3_ oxidation, and NO_*x*_ reduction on the CaO surface. In the absence of O_2_, CaO can catalyze NH_3_ decomposition to N_2_ [12,13]. CaO also catalyzes reactions between NH_3_ and NO in an O_2_-free atmosphere [5,14]. When O_2_ is present, these reactions do not apply, and the main effect of CaO is to catalyze NH_3_ oxidation with high NO selectivity [15,16].

Actually, gas-phase reactions coexist with the gas-solid catalytic reactions of the SNCR process in cement precalciners. However, previous research has only focused on the gas-solid catalytic reactions, not how these reactions interact with the gas-phase SNCR process. In addition, the temperature and gas conditions used in previous research did not match the conditions in cement precalciners. As a result, it is unclear how CaO affects overall NO_*x*_ reduction efficiency within the temperature range and under the variable gas conditions of a cement precalciner. Thus, the present work attempts to investigate the influence of CaO on NO_*x*_ reduction by SNCR with NH_3_ under variable gas compositions, using a fixed-bed reactor. Both gas-phase and gas-solid catalytic reactions were considered. The influences of gas compositions on the effect of CaO were also studied by varying NH_3_, H_2_O, O_2_, and CO contents. Pathways for the effect of gas compositions on NO_*x*_ reduction in CaO-containing SNCR process were proposed.

## 2. Materials and Methods

### 2.1. Experimental Setup

**Figure 1:**
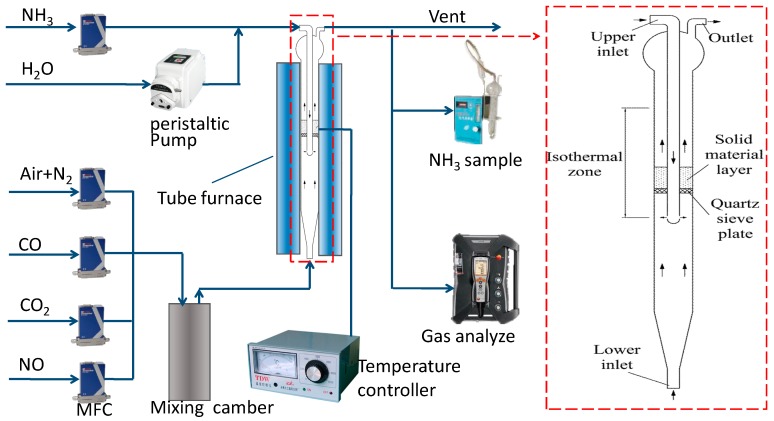
Schematic diagram of the experimental setup.

The experimental setup consists of a gas feeding unit, a high-temperature fixed bed experimental system, and gas analytical instruments. A schematic diagram is shown in Figure 1.

A quartz cylinder with a 25 mm inner diameter, 1100 mm length and two gas inlets was used as the SNCR reactor. A quartz sieve was correctly plated at the constant temperature zone of the electric furnace to support Ca-based particles. The inner temperature of the reaction zones was detected online by a thermal couple. The simulated cement precalciner gas stream containing N_2_, NO, CO, CO_2_ and air was introduced into the reactor from the lower inlet, the secondary stream with NH_3_ (diluted by N_2_) and water were fed into the reactor from the upper inlet. The two streams passed through respective preheating zones, and were then mixed in the reaction zones; the gas outlet was at the top of the reactor. The distance between the stream mixing point and the sieve plate was 50 mm.

### 2.2. Samples

CaO particles were prepared by decomposing analytical-grade CaCO_3_ with a particle size of 38–45 μm. In the experiments without CaO, SiO_2_ particles with a size of 38–45 μm were used as the background solid material due to its sluggishness. Prior to the experiments, SiO_2_ or CaCO_3_ was introduced into the reactor, distributed uniformly on the sieve plate, and then a preheating process was carried out: (1) The heating rate was set to 15 °C min^−1^ and the solid materials were gradually heated from room temperature. Meanwhile, the air flow rate was set to 0.1 dm^3^ min^−1^ to take away the CO_2_ generated from CaCO_3_ calcinations. During this stage, the sintering of the solid particles proceeded gradually; (2) When the temperature of the solid material was about 850 °C, the air-flow rate was set to 0.5 dm^3^ min^−1^; (3) When the temperature reached 1000 °C, the flow rate was set to 2 dm^3^ min^−1^. This stage lasted for 30 min; (4) The heating process was stopped, and the solid materials were cooled down to the target experimental temperature in N_2_ atmosphere. After the preheating process, CaCO_3_ completely decomposed to CaO, and the particle materials were sintered to a porous block structure to avoid being blown out by the gas stream.

The properties of the CaO samples are listed in Table 1.

### 2.3. Experimental Methods

The experiments were performed at atmospheric pressure. The basic cement precalciner gas was composed of 10% O_2_, 20% CO_2_, 3% H_2_O, 4000 ppm CO, 400 ppm NO, and 400 ppm NH_3_ with N_2_ used as balance gas, and the total flow rate was 2 dm^3^ min^−1^. The effects of both CaO amount and basic gas composition on NO_*x*_ reduction by SNCR were investigated. The concentrations of O_2_, CO, NO, and NO_2_ were monitored by using a Testo 350-pro flue gas analyzer. NH_3_ slip was sampled by a bubble column absorber containing dilute sulfuric acid solution and then analyzed by Nessler’s reagent spectrophotometry.

NO_*x*_ reduction efficiency, abbreviated as NO_*x*_ reduction, is measured based on inlet and outlet NO_*x*_ concentration. The temperature window is defined as the temperature region in which the NO_*x*_ reduction efficiency exceeds 80% of the maximum value. The NO_*x*_ reduction efficiency is defined as:

(1) $${\mathrm{NO}}_{x}\;\mathrm{reduction}=\frac{C_{{\mathrm{NO}}_{x},\mathrm{in}}-C_{{\mathrm{NO}}_{x},\mathrm{out}}}{C_{{\mathrm{NO}}_{x},\mathrm{in}}}\times 100\%$$

where $C_{{\mathrm{NO}}_{x},\mathrm{in}}$ and $C_{{\mathrm{NO}}_{x},\mathrm{out}}$ represent the inlet and outlet NO_*x*_ concentrations, respectively.

## 3. Results

### 3.1. Effect of CaO on SNCR

#### 3.1.1. Effect of CaO Amount on NO_x_ Reduction

**Figure 2:**
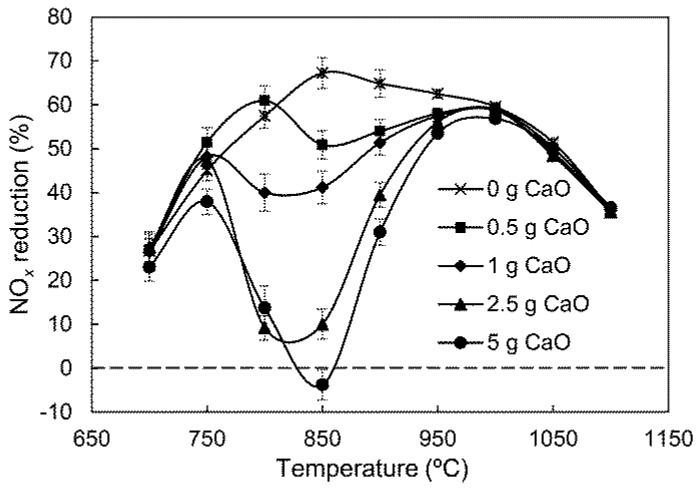
Effect of CaO amount on NO_*x*_ reduction by SNCR.

The effect of CaO amounts on NO_*x*_ reduction by SNCR was studied under the basic gas compositions, and the results are illustrated in Figure 2.

As shown in Figure 2, the NO_*x*_ reduction curve without CaO is a unimodal shape. The maximum reduction efficiency of approximately 68% occurs at 850 °C. The temperature window of SNCR is 800–1050 °C. After CaO is added into the reactor, the NO_*x*_ reduction efficiencies between 800 °C and 1000 °C decrease significantly, and these curves take on a bimodal shape. As CaO increases from 0.5 g to 5 g, the low-temperature peak value of NO_*x*_ reduction drops from 61% to 38% with the corresponding temperature change from 800 °C to 750 °C. The high-temperature peak value, which is approximately 58% at 1000 °C, is hardly influenced by CaO content. The trough value of NO_*x*_ reduction at 800–850 °C drops sharply with the increase of CaO content, and even a negative reduction efficiency, corresponding to the CaO of 5 g, appears at 850 °C.

As discussed in detail in our previous study [8], without CaO addition, the final NO_*x*_ reduction efficiency by SNCR is determined by the competition between gas-phase NO_*x*_ reduction and gas-phase NH_3_ oxidation processes:

(2) $$4\mathrm{NO}+4{\mathrm{NH}}_{3}+O_{2}\to 4N_{2}+6H_{2}O$$

(3) $$4{\mathrm{NH}}_{3}+5O_{2}\to 4\mathrm{NO}+6H_{2}O$$

(4) $$4{\mathrm{NH}}_{3}+3O_{2}\to 2N_{2}+6H_{2}O$$

In a CaO-containing atmosphere, reactions (3) and (4) also occur catalytically on the CaO surface, which can lead to extra NO_*x*_ formation, and may be responsible for the negative NO_*x*_ reduction efficiency at 850 °C in the 5 g CaO group of Figure 2.

#### 3.1.2. Effect of CaO on NH_3_ Oxidation

**Figure 3:**
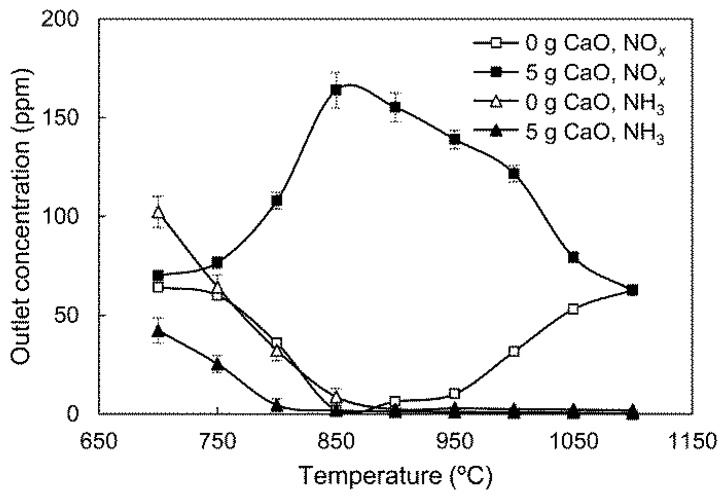
Effect of CaO amount on the oxidation of NH_3_ to NO_*x*_.

To investigate catalytic NH_3_ oxidation on the CaO, experiments were conducted under the basic gas compositions without NO. The results are shown in Figure 3.

Figure 3 demonstrates that NH_3_ can be oxidized to NO_*x*_ by gas-phase reactions. In the 0 g CaO group, the outlet NO_*x*_ concentration does not increase monotonically, as reported in other research [14], when temperature rises from 700 °C to 1100 °C. The relatively high level of outlet NO_*x*_ at 700–800 °C can be attributed to the high concentration of CO in the gas stream. As stated in our previous study [8], CO can enhance the conversion of NH_3_ to NH_2_ and NH at low temperatures, thus promoting gas-phase NH_3_ oxidation. As temperature rises, CO is gradually consumed by O_2_ in the preheating zone of the reactor, which inhibits the oxidation of NH_3_ to NO. When the temperature goes above 950 °C, the conversion of NH_3_ to NH is accelerated without CO, raising the outlet NO_*x*_ concentration. In the 5 g CaO group, the relationship of outlet NO_*x*_ concentration with temperature is almost opposite from the group with no CaO. As temperature rises, the NO_*x*_ concentration increases to its maximum at 850 °C and then gradually declines. There are two types of reaction process involving NH_3_ in the 5 g CaO group, namely, gas-solid catalytic NH_3_ oxidation and gas-phase NH_3_ conversion. For gas-solid catalytic NH_3_ oxidation, the initial and rate-determining step is the H-abstraction of adsorbed NH_3_ [17,18]:

(5) $${\mathrm{NH}}_{3(g)}\to {\mathrm{NH}}_{3(\mathrm{ad})}$$

(6) $${\mathrm{NH}}_{3(\mathrm{ad})}\to {\mathrm{NH}}_{2(\mathrm{ad})}+H^{+}+e$$

Then, deeper H-abstraction might occur with NH_2(ad)_, forming NH_(ad)_, generating NO:

(7) $${\mathrm{NH}}_{2(\mathrm{ad})}\to {\mathrm{NH}}_{\left(\mathrm{ad}\right)}+H^{+}+e$$

(8) $${\mathrm{NH}}_{\left(\mathrm{ad}\right)}+O_{\left(\mathrm{lattice}\right)}\to {\mathrm{HNO}}_{\left(\mathrm{ad}\right)}$$

(9) $${\mathrm{HNO}}_{\left(\mathrm{ad}\right)}\to \mathrm{NO}+H^{+}+e$$

In the presence of O_2_ and NO, NH_2(ad)_ can reduce adsorbed NO to form N_2_:

(10) $${\mathrm{NH}}_{2(\mathrm{ad})}+{\mathrm{NO}}_{\left(\mathrm{ad}\right)}\to {\mathrm{NH}}_{2}{\mathrm{NO}}_{\left(\mathrm{ad}\right)}$$

(11) $${\mathrm{NH}}_{2}{\mathrm{NO}}_{\left(\mathrm{ad}\right)}\to N_{2}+H_{2}O$$

As such, the NO_*x*_ selectivity of catalytic NH_3_ oxidation is determined directly by the proportion of NH_2(ad)_ involved in reactions (7) and (10).

For gas-phase NH_3_ oxidation and NO_*x*_ reduction, the rate-determining step is the conversion of NH_3_ to NH_2_ [19]:

(12) $${\mathrm{NH}}_{3}+O\to {\mathrm{NH}}_{2}+\mathrm{OH}$$

(13) $${\mathrm{NH}}_{3}+\mathrm{OH}\to {\mathrm{NH}}_{2}+H_{2}O$$

Figure 3 also shows that the outlet NH_3_ concentration with CaO addition is lower than that without CaO below 850 °C. This indicates that reactions (5) and (6) proceed faster than reactions (12) and (13) below 850 °C. Because most NH_3_ is oxidized in the gas-solid reaction process, the NO_*x*_ concentration is mainly affected by the NO_*x*_ selectivity of catalytic NH_3_ oxidation. This leads to the escalation of outlet NO_*x*_ concentrations up to 850 °C. According to the kinetic parameters in previous research, when the temperature rises from 700 °C to 1100 °C, the rate constant of catalytic NH_3_ oxidation varies insignificantly [5], while the rate constants of gas-phase NH_3_ conversion increase significantly [19]. As a result, above 850 °C, the gas-phase NH_3_ conversion rate gradually surpasses the catalytic NH_3_ oxidation rate. More NH_3_ is oxidized in gas-phase reaction processes with a low NO_*x*_ selectivity, diminishing the outlet NO_*x*_ concentration. When the temperature rises to 1100 °C, the gas-phase NH_3_ conversion is fast enough to prevent NH_3_ involvement in catalytic reactions, and almost all NH_3_ is consumed by the gas-phase process. In this condition, the catalytic NH_3_ oxidation on the CaO surface can be ignored. When NO is introduced into the gas stream, NH_3_ consumption by the gas-phase NO_*x*_ reduction process further decreases the NH_3_ in the catalytic oxidation. This narrows the temperature range with significant CaO effect to 750–1000 °C.

### 3.2. Influences of Gas Compositions on the Effect of CaO

#### 3.2.1. Influence of Initial NH_3_ Concentration

**Figure 4:**
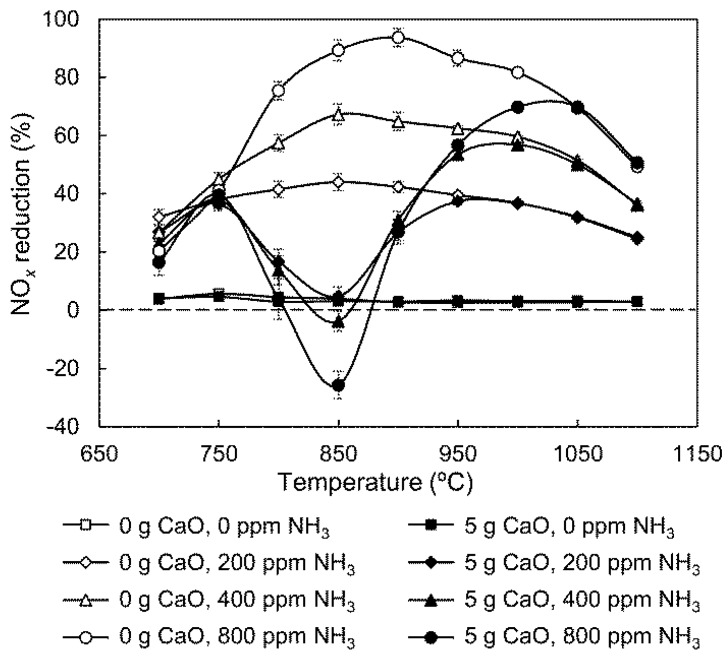
Effect of initial NH_3_ concentration on NO_*x*_ reduction.

In Figure 2, there is a slight increase in NO_*x*_ reduction efficiency at 700–800 °C in the 0.5 g CaO group compared with the group with no CaO. This improvement may be related to the NH_3_ concentration involved in gas-phase NH_3_ oxidation and NO_*x*_ reduction. To verify this, the effect of initial NH_3_ concentrations on SNCR was investigated, and the results are shown in Figure 4.

The NO_*x*_ reduction efficiency without CaO increases with initial NH_3_ concentration above 750 °C. However, there is an adverse result below 750 °C, suggesting that the lower initial NH_3_ concentration improves NO_*x*_ reduction at low temperatures. Therefore, when 0.5 g CaO is added, the NH_3_ involved in gas-phase NH_3_ oxidation and NO_*x*_ reduction decreases due to NH_3_ consumption through catalytic reactions, resulting in a slight increase in NO_*x*_ reduction efficiency at 700–800 °C.

It can also be seen from Figure 4 that increasing the initial NH_3_ concentration results in lower NO_*x*_ reduction efficiencies at 750–900 °C when CaO is added. At this temperature, gas-solid catalytic NH_3_ oxidation is more sensitive to NH_3_ concentration changes than gas-phase SNCR process. The initial NH_3_ concentration has a minor influence on the low-temperature peak value of NO_*x*_ reduction, but significantly augments the high-temperature one. Conversely, as shown in Figure 2, CaO content affects the low-temperature peak value notably, but has almost no effect on the high-temperature one. In an actual SNCR operation in a cement precalciner, NH_3_ addition is more controllable than CaO, so the temperature range of 950–1050 °C is more favorable for obtaining high reduction efficiency.

#### 3.2.2. Influence of H_2_O

**Figure 5:**
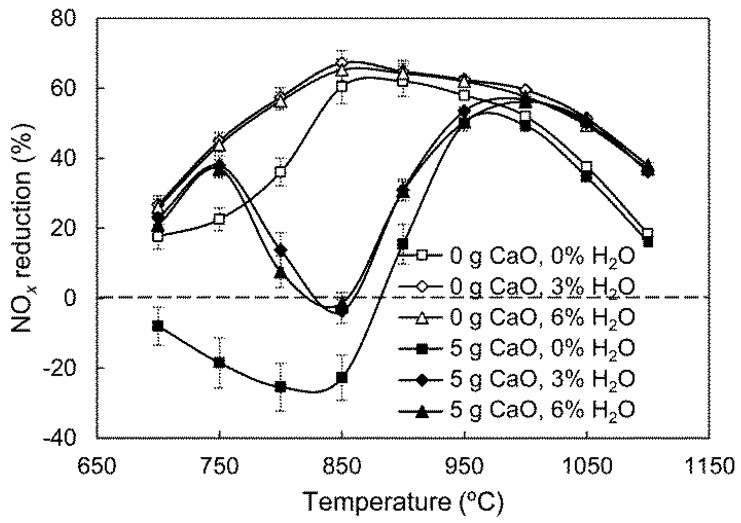
Influence of H_2_O on the effect of CaO.

The influence of H_2_O contents on the effect of CaO was investigated, and the results are shown in Figure 5.

Figure 5 shows that when no CaO is present, H_2_O has a positive effect on gas-phase SNCR process, which was discussed in our previous research [8]. In the 5 g CaO group, the NO_*x*_ reduction efficiency without H_2_O remains negative up to approximately 870 °C. When H_2_O is added, the efficiency increases significantly and the negative effect of CaO on NO_*x*_ reduction is suppressed to a certain extent. This may be because adding H_2_O stimulates gas-phase NH_3_ conversion, decreasing NH_3_ involved in catalytic oxidation. Furthermore, H_2_O can compete for active sites, adsorbing NH_3_ on the CaO surface, and thus inhibiting subsequent catalytic NH_3_ oxidation [16,20]. Therefore, H_2_O addition benefits NO_*x*_ reduction not only in a gas-phase reaction system, but also in a CaO-containing atmosphere.

#### 3.2.3. Influence of O_2_

**Figure 6:**
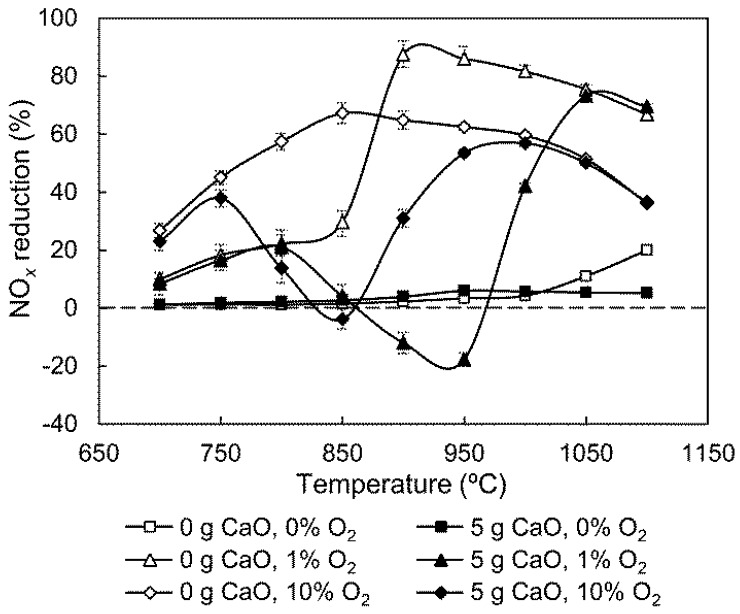
Influence of O_2_ on the effect of CaO.

O_2_ content is not uniform in cement precalciners, and generally ranges from 0% to 13%. Figure 6 shows the influence of O_2_ concentrations on the effect of CaO on SNCR.

As shown in Figure 6, the results without CaO coincide with our previous research. With CaO addition, when O_2_ content is reduced from 10% to 1%, the low-temperature peak, the trough, and the high-temperature peak of NO_*x*_ reduction curve shift to the higher temperatures of 800 °C, 950 °C, and 1050 °C, respectively. In addition, the trough value drops to a more negative one from −4% to −18%. This result suggests that decreasing O_2_ content shifts the temperature region in which CaO significantly impacts NO_*x*_ reduction from 750–1000 °C to the higher value range of 800–1050 °C, which shows that the gas-phase SNCR process is very susceptible to the change of O_2_ content, while the variation of O_2_ content between 1% and 10% fails to influence catalytic NH_3_ oxidation [11]. Hence, when O_2_ content decreases, the SNCR process slows and requires higher temperatures to proceed fast enough to dominate the whole process. As NO_*x*_ selectivity of catalytic NH_3_ oxidation rises with temperature, the minimum NO_*x*_ reduction efficiency drops.

O_2_ is necessary for the SNCR process, so when O_2_ is not present, the NO_*x*_ reduction efficiency without CaO is trivial below 1000 °C. The slight efficiency increase above 1000 °C is due to H radicals, which are generated from H_2_O pyrolysis and help NH_3_ conversion [8]. In contrast, the NO_*x*_ reduction efficiency of the 5 g CaO group remains trivial, even at high temperatures. Although CaO can catalyze NO reduction by NH_3_ in the absence of O_2_ [5,14], the catalyst cannot restore its activity without O_2_ reoxidation [18]. On the other hand, the competitive adsorption of H_2_O by CaO not only inhibits the catalytic NH_3_ + NO reaction, but also reduces gaseous H radicals. This weakens gas-phase NO_*x*_ reduction in the absence of O_2_. Therefore, it is not possible to reduce NO_*x*_ with NH_3_ in an O_2_-free zone in a cement precalciner.

#### 3.2.4. Influence of CO

**Figure 7:**
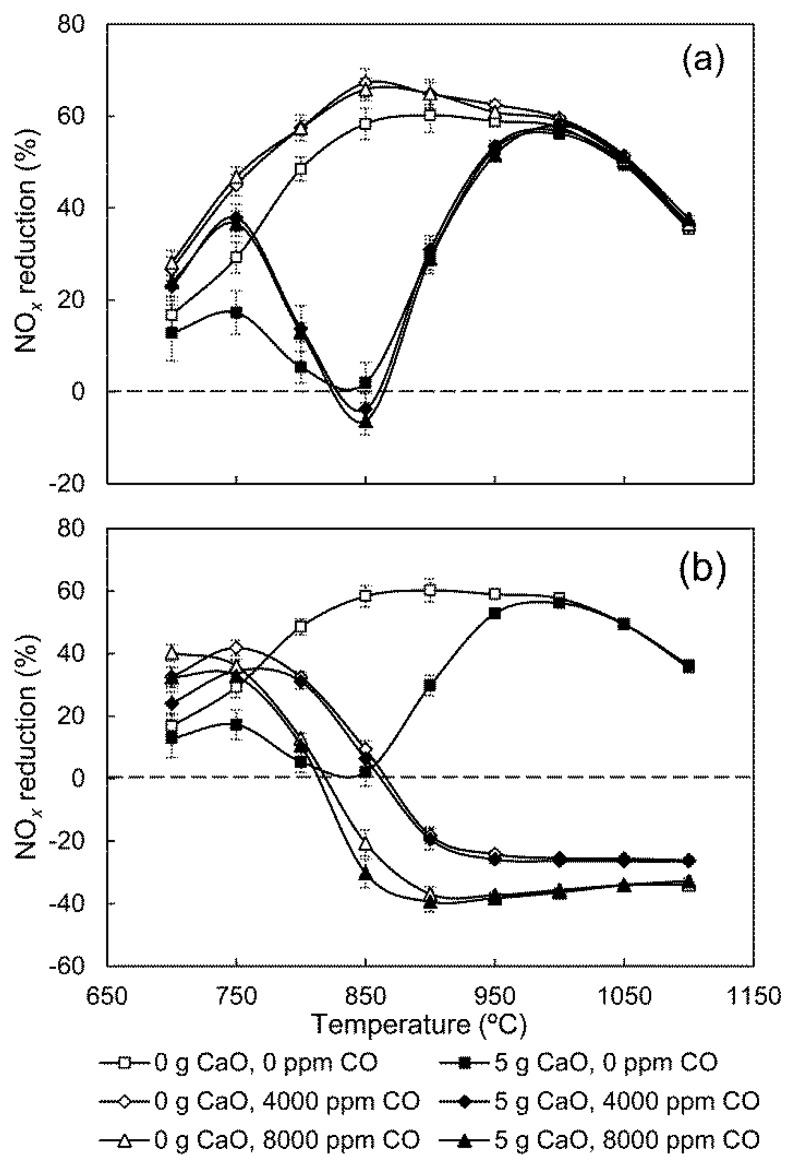
Influence of CO concentrations on the effect of CaO (**a**) when CO is introduced into the reactor with the main stream; and (**b**) when CO is introduced into the reactor with the secondary stream.

CO is an important constituent of flue gas in a cement precalciner. Similar to O_2_, it varies with location and operation conditions. It has been reported that CaO can catalyze NO reduction by CO, but the catalysis becomes negligible in the presence of either O_2_ or a high concentration of CO_2_ [21]. Previous research has investigated the influence of CO on gas-phase NO_*x*_ reduction [8,22,23]; however, it remains unclear how CO influences the effect of CaO on NO_*x*_ reduction with NH_3_. Therefore the influence of CO concentrations in a multi-phase reaction system was studied, and the results are seen in Figure 7.

Figure 7a shows an improvement of gas-phase NO_*x*_ reduction when adding CO at low temperatures. In the 5 g CaO group, the increase of CO content leads to a slight decrease of NO_*x*_ reduction efficiency between 820 °C and 900 °C, implying that the effect of CaO is enhanced. This may be attributed to H_2_O consumption by CO [24]:

(14) $$\mathrm{CO}+\mathrm{OH}\to {\mathrm{CO}}_{2}+H$$

(15) $$H+H_{2}O\to \mathrm{OH}+H_{2}$$

This series of reactions weakens the inhibitory effect of H_2_O on catalytic NH_3_ oxidation, engaging more NH_3_ in catalytic oxidation, leading to incremental NO_*x*_ generation. During the experiments, it was observed that the outlet CO concentration with the addition of CaO was lower than that without CaO under the same conditions. This is because CaO can catalyze CO oxidation [14], which suggests that there exist active sites adsorbing CO onto the CaO surface. If the active sites for CO are the same as those for NH_3_, CO should inhibit the negative effect of CaO on NO_*x*_ reduction similarly to H_2_O. However, the results suggest that the active sites adsorbing CO and NH_3_ are distinct, and that CO does not directly influence catalytic NH_3_ oxidation. Therefore, the only reason that adding CO would enhance NO_*x*_ reduction efficiency below 820 °C in the 5 g CaO group is that CO can promote the gas-phase SNCR process. The influence of CO can be ignored above 1000 °C in the group with no CaO, due to CO consumption by O_2_ in the reactor’s preheating zone. In the 5 g CaO group, because CO oxidation is promoted by CaO, the influence of CO is already negligible at 900 °C.

As can be seen from Figure 7b, when CO is introduced into the reactor with the secondary stream, NH_3_ is actually injected into an O_2_-free atmosphere with a high CO content before mixing with the stream containing O_2_. In this case, a large amount of NH_3_ is converted to NH_2_ and NH radicals, which shifts the temperature window of SNCR to lower values and causes negative NO_*x*_ reduction efficiencies at high temperatures [8]. Additionally, except that the NO_*x*_ reduction efficiency of 5 g CaO group is a little lower than that of the 0 g CaO group below 800 °C, there is little difference between the results of the two groups with CO addition. This means that the negative effect of CaO on NO_*x*_ reduction is almost completely suppressed. Consequently, it can be inferred that NH_2_ and NH radicals generated from gas-phase NH_3_ conversion cannot be adsorbed by CaO to join in the catalytic reactions. The catalytic oxidation of NH_3_ must be initiated by the adsorption of molecular NH_3_.

Although the injection method in Figure 7b can suppress the negative effect of CaO on NO_*x*_ reduction, NH_3_ is apt to be oxidized to NO_*x*_ via gas-phase reactions. Therefore, in an actual SNCR operation for cement precalciners, NH_3_ should not be injected into an O_2_-free area with high CO and then mixed with an O_2_-containing gas stream.

## 4. Discussion

Homogeneous reactions (gas-phase) play a predominant role in non-CaO SNCR in cement precalciners, and the rate-determining step is the conversion of NH_3_ to NH_2_. For heterogeneous catalytic reactions (gas-solid), the rate-determining step is the H-abstraction of adsorbed NH_3_ [17,18]. CaO has an inhibitory effect on the SNCR process, especially in the middle temperature zone of 750–1000 °C; the CaO contents are closely related to the inhibitory effect of CaO. CaO can catalyze NH_3_ decomposition. Moreover, CaO catalyzed NH_3_ oxidation in the presence of O_2_, which can lead to extra NO_*x*_ formation.

**Figure 8:**
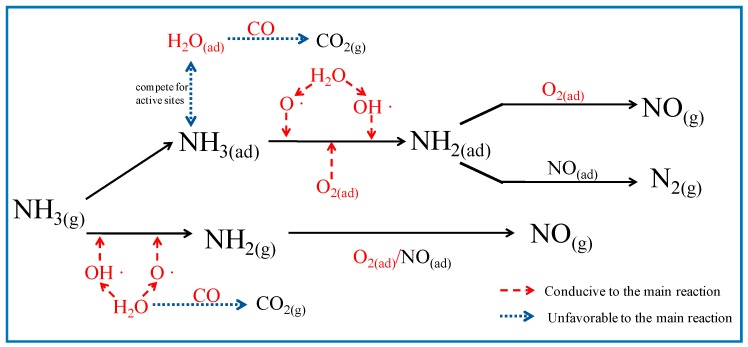
Proposed pathways for the effect of gas compositions on NO_*x*_ reduction in CaO-containing SNCR process.

The effects on NO_*x*_ reduction efficiency in the presence of CaO with the increase of H_2_O, O_2_, and CO concentration are different. The addition of H_2_O benefits NO_*x*_ reduction in homogeneous reactions, and suppresses the negative effect of CaO on NO_*x*_ reduction to a certain extent. The thermal decomposition of H_2_O results in the regeneration of O and OH radicals [8]; these radicals then stimulate gas-phase NH_3_ conversion, decreasing the NH_3_ involved in catalytic oxidation. Furthermore, H_2_O can compete for NH_3_ active sites on the CaO surface, and thus reducing the occurrence of heterogeneous reactions; O_2_ is indispensable for NO_*x*_ reduction by SNCR, as O_2_ is the primary reactant of NO production from the reaction of NH_2_ with O_2_ in the CaO-containing SNCR process, which causes a negative effect of CaO on NO_*x*_ reduction. CO leads to a slight decrease of NO_*x*_ reduction efficiency between 820 °C and 900 °C, and enhances the effect of CaO, which may be attributed to H_2_O consumption by CO [24]. The influence of CO is already negligible above 900 °C. The results of this study could provide a reference for engineering applications for optimizing NO_*x*_ reduction processes by NH_3_-SNCR in cement precalciners. A pathway for the effect of gas compositions on NO_*x*_ reduction in CaO-containing SNCR processes, based on conclusions derived from the experimental results and acknowledged reactions, is given in Figure 8.

## 5. Conclusions

CaO in a cement precalciner can inhibit NO_*x*_ reduction by the SNCR process between 750 °C and 1000 °C due to the catalytic oxidation of NH_3_ to NO on the CaO surface. When CaO is added, NO_*x*_ reduction efficiency follows a bimodal distribution against temperature. The low-temperature peak changes with CaO content, while the high-temperature peak is hardly influenced by CaO content. The trough value between the two peaks decreases significantly as CaO increases. With a high CaO content, the trough value of NO_*x*_ reduction at 850 °C is even negative. In CaO-containing cement precalciners, gas-phase NH_3_ conversion coexists with gas-solid catalytic NH_3_ oxidation. Below 750 °C, the NO_*x*_ selectivity of catalytic NH_3_ oxidation is low, so CaO does not significantly impact NO_*x*_ reduction efficiency. Above 1000 °C, the gas-phase NH_3_ conversion proceeds fast enough to prevent NH_3_ from being oxidized catalytically. Although NO_*x*_ reduction performance appears to improve by adding CaO at low temperatures, the improvement is slight, and likely results from the loss of NH_3_ involved in gas-phase NH_3_ conversion.

NH_3_, H_2_O, O_2,_ and CO influences the effect of CaO by affecting gas-phase NH_3_ conversion, or gas-solid catalytic NH_3_ oxidation, or both processes. Although increasing NH_3_ concentration is advantageous to NO_*x*_ reduction, increasing NH_3_ results in a decrease in NO_*x*_ reduction efficiency at 750–900 °C when CaO is added. H_2_O can significantly suppress the negative effect of CaO on NO_*x*_ reduction. As O_2_ content decreases from 10% to 1%, the temperature region for a significant CaO effect shifts higher. With the addition of CO, the effect of CaO is reduced below 820 °C, while it is slightly enhanced at approximately 850 °C. When NH_3_ is injected into an O_2_-free atmosphere with a high CO content, it is apt to be oxidized to NO_*x*_ after mixing with an O_2_-containing stream. In this case, the effect of CaO on NO_*x*_ reduction is almost eliminated.

Considering the effect of CaO on NO_*x*_ reduction under variable gas compositions, it is recommended that NH_3_ be injected into the O_2_-containing area with a low CaO concentration in the temperature range of 950–1100 °C to obtain a high NO_*x*_ reduction efficiency by SNCR in a cement precalciner.

## Figures and Tables

**Table 1:** Properties of pretreated CaO sample.

Morphology	Porous Block
Average particle diameter (μm)	38–45
Specific surface area (m^2^ g^−1^)	37.162
Specific pore volume (cm^3^ g^−1^)	0.091
Average pore diameter (nm)	11.302
Density (g cm^−3^)	3.35
